# Living on the Edge: Physiological and Kinetic Trade-Offs Shape Thermal Tolerance in Intertidal Crabs From Tropical to Sub-Antarctic South America

**DOI:** 10.3389/fphys.2020.00312

**Published:** 2020-04-24

**Authors:** Samuel Coelho Faria, Adalto Bianchini, Mariana Machado Lauer, Ana Lúcia Ribeiro Latorre Zimbardi, Federico Tapella, Maria Carolina Romero, John Campbell McNamara

**Affiliations:** ^1^Departamento de Biologia, Faculdade de Filosofia, Ciências e Letras de Ribeirão Preto, Universidade de São Paulo, Ribeirão Preto, Brazil; ^2^Instituto de Ciências Biológicas, Universidade Federal do Rio Grande, Rio Grande, Brazil; ^3^Departamento de Química, Faculdade de Filosofia, Ciências e Letras de Ribeirão Preto, Universidade de São Paulo, Ribeirão Preto, Brazil; ^4^Centro Austral de Investigaciones Científicas, CADIC-CONICET, Ushuaia, Argentina; ^5^Centro de Biologia Marinha, Universidade de São Paulo, São Sebastião, Brazil

**Keywords:** evolutionary physiology, thermal adaptation, critical limits, oxygen consumption, lactate, LDH, Brachyura

## Abstract

Temperature is an important abiotic factor that drives the evolution of ectotherms owing to its pervasive effects at all levels of organization. Although a species’ thermal tolerance is environmentally driven within a spatial cline, it may be constrained over time due to differential phylogenetic inheritance. At the limits of thermal tolerance, hemolymph oxygen is reduced and lactate formation is increased due to mismatch between oxygen supply and demand; imbalance between enzyme flexibility/stability also impairs the ability to generate energy. Here, we characterized the effects of lower (LL_50_) and upper (UL_50_) critical thermal limits on selected descriptors of aerobic and anaerobic metabolism in 12 intertidal crab species distributed from northern Brazil (≈7.8°S) to southern Patagonia (≈53.2°S), considering their phylogeny. We tested for (i) functional trade-offs regarding aerobic and anaerobic metabolism and LDH kinetics in shaping thermal tolerance; (ii) influence of shared ancestry and thermal province on metabolic evolution; and (iii) presence of evolutionary convergences and adaptive peaks in the crab phylogeny. The tropical and subtropical species showed similar systemic and kinetic responses, both differing from the sub-Antarctic crabs. The lower UL_50_’s of the sub-Antarctic crabs may reflect mismatch between the evolution of aerobic and anaerobic metabolism since these crabs exhibit lower oxygen consumption but higher lactate formation than tropical and subtropical species also at their respective UL_50_’s. LDH activity increased with temperature increase, while K_m_^Pyr^ remained fairly constant; catalytic coefficient correlated negatively with thermal niche. Thermal tolerance may rely on a putative evolutionary trade-off between aerobic and anaerobic metabolism regarding energy supply, while temperature compensation of kinetic performance is driven by thermal habitat as revealed by the LDH affinity/efficiency equilibrium. The overall physiological evolution revealed two homoplastic adaptive peaks in the sub-Antarctic crabs with a further shift in the tropical/subtropical clade. The physiological traits at UL_50_ have evolved in a phylogenetic manner while all others were more plastic. Thus, shared inheritance and thermal environment have driven the crabs’ thermal tolerance and metabolic evolution, revealing physiological transformations that have arisen in both colder and warmer climes, especially at higher levels of biological organization and phylogenetic diversity.

## Introduction

Biological systems are thermodynamically open, owing to the continuous flux of energy and matter with the surrounding environment (Cannon, 1929; Recordati and Bellini, 2004). Temperature is the main abiotic factor that drives the biogeographical evolution of ectotherms (Pörtner, 2002; Johns and Somero, 2004; Somero, 2004, 2010; Dong et al., 2008; Stevens et al., 2010; Tomanek and Zuzow, 2010) since it affects all levels of organization, from the subcellular to the systemic, including the intact organism. The deleterious effects of temperature often exceed the regulatory capability of an organism’s homeostatic mechanisms, and thus limit the distribution of life, imposing constraints on biogeographical distributions.

In aquatic decapod crustaceans at usual habitat temperatures, the capacity for oxygen uptake by ventilation and its delivery by circulation matches the biochemical demands of the tissues for oxygen: the fully oxygenated hemolymph thus can sustain optimal aerobic metabolism in the tissues (Pörtner and Gutt, 2016; Verberk et al., 2016). However, at the critical thermal limits, hemolymph oxygen levels are reduced due to the mismatch between oxygen supply and demand, imposing limitations on temperature tolerance (Pörtner, 2002). This limitation to systemic oxygen availability creates a hypoxic state, which is unable to sustain tissue demand, leading to anaerobic metabolism at the critical thermal limits as a consequence of elevated oxygen demand or insufficient aerobic capacity (Giomi and Pörtner, 2013). The recruitment of chaperone molecules and anti-oxidant defenses may extend survival, albeit with reduced performance at the molecular level, reverberating at the whole animal level and leading to death. This unifying principle known as “oxygen and capacity limitation of thermal tolerance” (OCLTT) characterizes sensitivity to thermal stress in aquatic invertebrates (Pörtner and Gutt, 2016; Verberk et al., 2016). During aerial exposure, however, air-breathers and amphibious species can attain higher hemolymph oxygen concentrations and increase oxygen diffusion, which may enhance their thermal tolerance by extending aerobic scope (Giomi et al., 2014).

To illustrate, the spider crab *Maja squinado* exhibits highly oxygenated hemolymph between 8 and 17°C, showing decreased oxygen partial pressures below 8°C and increased hemolymph lactate above 17°C (Frederich and Pörtner, 2000). The shore crabs *Carcinus maenas* and *Cancer irroratus* also accumulate hemolymph lactate at temperatures above 32 and 28°C, respectively, after direct transfer from 12°C (Jost et al., 2012). The transition to a predominantly anaerobic metabolism in the lobster *Homarus americanus* takes place gradually and continuously, accompanying a progressive increase in hemolymph lactate, reaching a maximum at 30°C (Jost et al., 2012). Clearly, the limitation of oxygen supply to the tissues is linked to the onset of anaerobiosis due to a decreased capacity for oxygen uptake and transport. Further, patent patterns of increased oxygen demand and lactate formation are evident when temperature becomes life limiting.

Considering the subcellular environment, kinetic adjustments can result from oxygen- and capacity-limited thermal tolerance (Hochachka and Somero, 2002). Enzymes exhibit an inherent structural flexibility that guarantees their molecular function. However, they must also preserve the molecular rigidity that maintains their three-dimensional structure (Zavodszky et al., 1998). The balance between flexibility and stability in enzyme kinetics is temperature dependent, and is particularly evident with regard to lactate dehydrogenase (LDH) in vertebrate species. LDH converts pyruvate to lactate during anaerobic metabolism, reflecting the ability to produce energy under critical thermal conditions as predicted by the OCLTT principle (Zakhartsev et al., 2004).

Comparative studies of LDH have explored certain lineages of ectothermal vertebrates and invertebrates from various thermal niches, although a phylogenetic approach has only been implemented in the latter. In response to increased temperature, vertebrate kinetic patterns show decreased binding capacity and increased catalytic efficiency (Fields and Somero, 1998; Hochachka and Somero, 2002; Somero, 2003, 2004). LDH activity tends to increase at higher temperatures as a consequence of the augmented energy available for the conformational changes required during catalysis (Dunn et al., 1991; Fields, 2001; Hochachka and Somero, 2002). These kinetic properties of ectothermal vertebrate enzymes reflect a highly competent or occasionally a marginally functional LDH, depending on thermal regime. In contrast, in invertebrates, particularly exemplified by species of the anomuran genus *Petrolisthes*, LDH thermal stability is not affected by maximum habitat temperature, suggesting likely phylogenetic constraints on interspecific variability in LDH stability (Stillman and Somero, 2001).

**FIGURE 1:**
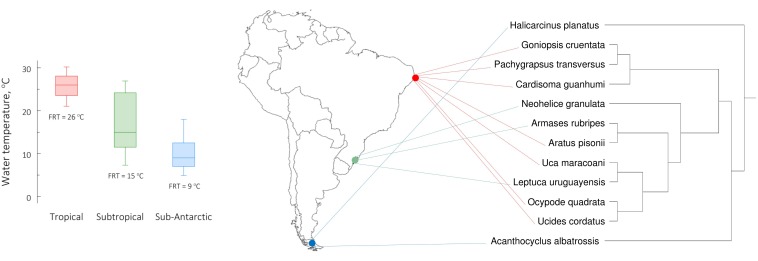
Mean, monthly, maximum and minimum surface seawater temperatures registered between 2009 and 2019 (National Oceanic and Atmospheric Administration [NOAA] and U.S. Department of Commerce, 2019) (*left panel*) at the northern and southern geographical limits, respectively, of each zoogeographical province along the eastern Atlantic coast of South America (*sensu* Boschi, 2000a). Surface seawater temperatures varied between 20.7 and 30.5°C for the Brazilian province, 7.0 and 27.5°C for the Argentinian province, and 4.0 and 18.0°C for the Magellanic province. The horizontal line within each box indicates the median temperature while the box boundaries show the interquartile values. Whiskers indicate the lowest and highest temperatures (range). Collecting site locations are indicated within each zoogeographical province (*middle panel*). The temperatures 26, 15, and 9°C represent the field reference temperatures (FRT) at which the crabs from each province were acclimated. The tropical species (*Aratus pisonii, Cardisoma guanhumi, Goniopsis cruentata, Ocypode quadrata*, *Pachygrapsus transversus, Uca maracoani*, and *Ucides cordatus*) were collected from the Brazilian province (northeastern Brazil, ≈7.8°S, 34.8°W, red). The subtropical species (*Armases rubripes, Neohelice granulata* and *Leptuca uruguayensis*) were collected from the Argentinian province (southern Brazil, ≈32.1°S, 52.1°W, green). The sub-Antarctic species (*Acanthocyclus albatrossis* and *Halicarcinus planatus*) were collected from the Magellanic province (southern Patagonia, ≈53.2°S, 67.2°W, blue). The phylogenetic relationships among the species investigated (*right panel*) were generated employing a maximum likelihood search method using the mitochondrial 16S ribosomal gene as a marker (see Faria et al., 2017).

Considering environments that offer a wide thermal landscape, the continental shelves of the Americas are divided into sixteen zoogeographic provinces. These have been defined based on “*part of the neritic zone with a relatively narrow range of temperatures where the fauna shows certain homogeneity*,” and particularly on oceanographic characteristics like water temperature and ocean currents (Boschi, 2000a,b; Boschi and Gavio, 2005). The eastern coast of South America embraces a significant thermal gradient and includes three provinces (Figure 1). The Brazilian province, between the mouth of the Orinoco river, Venezuela (9°N) and Cabo Frio, Rio de Janeiro, southeastern Brazil (23°S) (Briggs, 1974; Boschi, 2000a,b) in which mean surface water temperatures vary between 20.7 and 30.5°C (median = 26°C). The Argentinian province, between Cabo Frio and Rawson, Chubut, central Argentina (43–44°S) (Cooke, 1895; Boschi, 2000a), which exhibits temperatures between 7.0 and 27.5°C (median = 15°C). And the Magellanic province, between Rawson and Tierra del Fuego (55°S), Patagonian Argentina (Carcelles and Williamson, 1951; Balech, 1954; Boschi, 2000a,b) in which temperatures vary between 4.0 and 18.0°C (median = 9°C). These ranges represent the minimum and maximum mean monthly temperatures registered between 2009 and 2019 at the northern and southern geographical limits of each province (National Oceanic and Atmospheric Administration [NOAA] and U.S. Department of Commerce, 2019).

We have shown (Faria et al., 2017) that the micro-habitat temperatures (MHT) of some crab species from the Brazilian and Argentinian zoogeographical provinces are similar, but are higher than those of the Magellanic species. The upper (UL_50_) and lower (LL_50_) critical thermal limits of these crabs are subject to different environmental pressures, and exhibit unique evolutionary histories. The UL_50_ show phylogenetic signal and correlate with micro-habitat temperature, while the LL_50_ are more plastic and differ markedly among all provinces, revealing an asymmetrical evolutionary history of the critical thermal limits.

In the present study, we now investigate physiological and evolutionary patterns of thermal tolerance in this crab clade (Faria et al., 2017), characterizing the effects of critical thermal limits (LL_50_ and UL_50_) on selected descriptors of aerobic and anaerobic metabolism at the systemic and kinetic levels. Using 12 intertidal crab species distributed across the three thermal provinces from northern Brazil to southern Patagonia, we tested for: (i) the influence of shared ancestry and thermal province on metabolic evolution; (ii) the existence of functional trade-offs among aerobic and anaerobic metabolism and LDH kinetics in shaping thermal tolerance; and (iii) the presence of evolutionary convergences and adaptive peaks in the crab phylogeny. This study considers that species’ traits are linked to each other through space and time, embracing most dimensions of physiological evolution.

## Materials and Methods

### Crab Sampling and Laboratory Maintenance

Approximately 70 adult, intermolt, male or female crabs of each of the 12 species were collected during the morning, at low tide, from mangroves, salt marshes, and sandy or rocky beaches, at the ends of the summers of 2013 and 2014. Collection sites included the Brazilian, Argentinian and Magellanic zoogeographical provinces (Boschi and Gavio, 2005; Figure 1), which correspond roughly to the Tropical, Subtropical and sub-Antarctic physiographical zones.

In the laboratory, the crabs were maintained prior to experiments for 5 days in plastic boxes, each containing a thin layer of seawater (≈3 mm deep, 30 ‰S) to allow gill wetting, in incubators or in water baths (Fanem 347 DCG; Solab SL 224; Polystat 12002, Palmer Instrument Company) at the respective field reference temperatures (FRT) of each zoogeographical province (see above). These temperatures represent the median of the mean monthly minimum and maximum temperatures registered at the northern and southern geographical limits of each province (sensu Boschi, 2000a; National Oceanic and Atmospheric Administration [NOAA] and U.S. Department of Commerce, 2019). This protocol also minimized possible intrinsic physiological variability related to the species’ vertical positions within the intertidal habitat. The crabs were not fed during the experiments, and the seawater was replaced daily in the morning.

The tropical species (*Aratus pisonii* H. Milne Edwards, 1837; *Cardisoma guanhumi* Latreille, 1825; *Goniopsis cruentata* Latreille, 1803; *Ocypode quadrata* Fabricius, 1787; *Pachygrapsus transversus* Gibbes, 1850; *Uca maracoani* Latreille 1802–1803; and *Ucides cordatus* Linnaeus, 1763) were collected from beaches or mangroves on Ilha de Itamaracá or Itapissuma, Pernambuco (northeastern Brazil, ≈7.8°S, 34.8°W). They were held at the FRT of 26°C (Figure 1) on a 12 h light/12 h dark photoperiod at the Universidade Federal de Pernambuco (Recife, Brazil).

The subtropical species (*Armases rubripes* Rathbun, 1897; *Neohelice granulata* Dana, 1851; and *Leptuca uruguayensis* Nobili, 1901) were collected from beaches near Rio Grande, Rio Grande do Sul (southern Brazil, ≈32.1°S, 52.1°W). They were held at the FRT of 15°C (Figure 1) on a 14 h light/10 h dark photoperiod at the Universidade Federal do Rio Grande (Rio Grande, Brazil).

The sub-Antarctic representatives (*Acanthocyclus albatrossis* Rathbun, 1898; and *Halicarcinus planatus* Fabricius, 1775) were collected from stony beaches near Ushuaia, Tierra del Fuego (southern Argentina, ≈53.2°S, 67.2°W). They were held at the FRT of 9°C (Figure 1) on a 14 h light/10 h dark photoperiod, at the Centro Austral de Investigaciones Científicas, Consejo Nacional de Investigaciones Científicas y Técnicas (Ushuaia, Tierra del Fuego, Argentina).

### Critical Thermal Limits

Lower (LL_50_) and upper (UL_50_) critical thermal limits and microhabitat temperatures for each of the species evaluated here have been published previously (Table 1; Faria et al., 2017). Both limits define the temperatures at which “*the animal loses its ability to escape from conditions that will promptly lead to its death*” (Cowles and Bogert, 1944). At each limit, 50% of the crabs were unable to right themselves when placed in a supine position. This inability reflects the loss of key motor functions and/or potentially lethal, disorganized locomotory activity (Faria et al., 2017).

**TABLE 1:** Crab species collected from different zoogeographical provinces along the eastern Atlantic coast of South America, providing their microhabitat temperatures (MHT) and lower (LL_50_) and upper (UL_50_) critical thermal limits (all in °C) (modified from Faria et al., 2017).

**Species**	**Substrate**	**MHT**	**LL_50_**	**UL_50_**
**Brazilian province (Tropical)**				
*Aratus pisonii*	Mangrove trees	27.0 ± 0.6	12.8	36.9
*Cardisoma guanhumi*	Sandy-clay	26.2 ± 0.4	13.4	38.6
*Goniopsis cruentata*	Mangrove mud	28.2 ± 0.8	12.8	36.0
*Ocypode quadrata*	Sandy beaches	21.8 ± 0.8	13.8	36.2
*Pachygrapsus transversus*	Rocky shores	29.1 ± 1.1	13.8	36.2
*Uca maracoani*	Mangrove mud	36.3 ± 0.5	12.8	38.6
*Ucides cordatus*	Mangrove mud	22.1 ± 1.5	15.5	39.0
		**27.2 ± 1.4**	**13.6 ± 0.4**	**37.4 ± 0.5**
**Argentinian province (Subtropical)**				
*Armases rubripes*	Rocky shores	27.0 ± 1.2	8.7	36.1
*Neohelice granulata*	Sandy-clay	25.5 ± 0.7	6.5	36.7
*Uca uruguayensis*	Sandy beaches	29.3 ± 1.4	10.4	39.3
		**27.3 ± 0.6**	**8.5 ± 1.1**	**37.4 ± 1.0**
**Magellanic province (Sub-Antarctic)**				
*Acanthocyclus albatrossis*	Rocky shores	1.5 ± 0.1	−0.2	29.0
*Halicarcinus planatus*	Rocky shores	1.4 ± 0.1	−0.1	23.0
		**1.5 ± 0.1**	−**0.2 ± 0.1**	**26.0 ± 3.0**

*MHT data are the mean ± SEM (7 ≤ N ≤ 15). Data are independent of species’ vertical positions (Stillman and Somero, 2000) within their intertidal zones (pGLS, 0.77 ≤ F ≤ 3.9, 0.23 ≤ P ≤ 0.54). Bold values are the mean ± SEM for all species in each province.*

In brief, LL_50_ and UL_50_ were established (8 ≤ *N* ≤ 10 crabs per temperature and species) by direct transfer of the crabs to various lower or higher temperatures, respectively, from their respective FRT’s (26°C for the tropical, 15°C for the subtropical, and 9°C for the sub-Antarctic species). The crabs were examined for 30 s every 30 min during a 6 h period. This duration of exposure represents “*the mean emergence time of a crab at low tide”* (Faria et al., 2017) and constitutes the mean duration of exposure to aerial temperatures during low tide.

This experimental design, i.e., direct exposure from the respective FRT to the LL_50_ or UL_50_ of each species, enabled quantitative measurement of the critical thermal limits of each species, in line with Faria et al.’s (2017) protocol. It thus allowed direct comparison and straightforward correlations between the critical thermal limits and the physiological traits evaluated.

### Mass-Specific Oxygen Consumption

Individual crabs (10 ≤ *N* ≤ 15) were placed into acrylic respirometer chambers containing a 3 mm deep layer of seawater (30 ‰S), constructed specifically to accommodate each species with regard to diameter and volume. The respirometers were held in incubators or water baths (Fanem 347 DCG; Solab SL 224; Polystat 12002, Palmer Instrument Company) at the respective FRT of each species for a 12 h adjustment period prior to beginning the measurements. Each respirometer was coupled by plastic Crystal tubing to a multiplexing manometer and a gas analyzer (FoxBox-C Field Oxygen & Carbon Dioxide Analysis System, Sable Systems International Inc.), and perfused with an airflow of from 150 to 600 mL min^–1^, depending on respirometer volume, standardized for each species. The standardized airflow provided a baseline percentage of atmospheric oxygen (20.8% O_2_) in the absence of ambient pressure variation owing to the automated, barometric pressure compensation system.

After the 12 h adjustment period at the FRT, the respirometers were transferred to an incubator or water bath to measure O_2_ consumption at the LL_50_ or UL_50_ temperatures established previously for each species, using a stop-flow respirometric protocol (Steffensen, 1989). After a 4 h period of continuous airflow, the respirometers were sealed by closing their respective manometer valves for a ≈2 h period. The O_2_ respired by each crab in each respirometer was then measured by recording the decrease in percentage oxygen compared to the pre-established baseline reading while renewing the airflow during a 6 min period of flushing to the gas analyzer. Since measurements were conducted at the critical thermal limits, some crabs died during the measurements. Only the lowest O_2_ consumption measurements recorded from at least 5–7 crabs alive at the end of each experiment were used.

After each experiment, each crab was cryoanesthetized in a freezer and killed by immersion for 20 min in crushed ice. The crabs were then dried in an oven at 60°C for up to 72 h, and their dry masses were measured using an analytical balance (±1 mg precision).

Data acquisition software (Expedata, Sable Systems International Inc.) was used to calculate the integral of the area corresponding to the decrease in percentage oxygen (I_A_) in the flushed air. The individual mass-specific oxygen consumption rate (QO_2_) for each crab was obtained from the formula:

QO(mLOg2h-1)-12

 =I×Af(mLmin)-1/[100×m(g)×t(min)]×60

where I_A_ is oxygen percentage area; f, airflow rate (150–600 mL min^–1^ through each respirometer during the open phase); m, dry mass; t, duration of sealed phase. The factors “100” and “60” convert O_2_ percentage to O_2_ volume, and minutes to hours, respectively.

The effect of body mass on QO_2_ was removed by transforming the QO_2_ measurements into log_10_ values, which were then regressed against the respective dry masses. The residues of this regression were then employed, thus ensuring mass-free data.

### Extraction of Hemolymph and Abdominal Muscle Samples

Specimens (10 ≤ *N* ≤ 15) of each crab species were transferred from their respective FRT’s to their respective LL_50_ and UL_50_ temperatures in incubators or water baths. After a 6 h exposure period at each thermal limit, a 50 μL hemolymph sample was drawn through the arthrodial membrane at the base of the last pereiopod of the surviving crabs using a #27-5 gauge needle coupled to an insulin syringe. Approximately 50 mg of abdominal muscle were also dissected. All samples were frozen immediately in liquid nitrogen and transferred in dry ice to the Laboratory of Crustacean Physiology at the University of São Paulo in Ribeirão Preto where they were stored at −80°C until analyses could be performed.

### Hemolymph Lactate

Perchloric acid (6%) was added to each hemolymph sample (3:1, v/v), followed by centrifugation at 10,000 g for 5 min at 4°C. The supernatant was neutralized with 5 M K_2_CO_3_ (10:1, v/v) and re-centrifuged. For microplate reading, an aliquot of each sample was added to a buffer containing 0.5 M glycine, 0.4 M hydrazine and 10 mM EDTA at pH 9.0. 1 mM NAD^+^ (final concentration) and 20 U/mL LDH (final concentration) were added to each well, and the microplate was incubated at 25°C for 1 h in the dark and read at 340 nm (Bergmeyer and Bernt, 1974; Santos and Keller, 1993; Henry et al., 1994). The lactate concentration (mg L^–1^) was obtained from the formula:

Lactate concentration (mg L)-1

 ={[(A×0.3)⁢(6.22×0.874×0.015)]×3.4}×90.1

where A, Absorbance; 0.3, final fluid volume in each well; 6.22, molar extinction coefficient of NADH at 340 nm; 0.874, well optical path; 0.015, sample volume in each well; 3.4, sample dilution factor; and 90.1, conversion factor from mmol L^–1^ to mg L^–1^.

### Temperature Coefficient

The effect of temperature on QO_2_ (aerobic sensitivity) and on lactate concentration (anaerobic sensitivity) was expressed using the formula Q_10_ = (k_1_/k_2_)^10/(t1–t2)^, where k_1_ and k_2_ are the mass specific oxygen consumption rates at temperatures t_1_ and t_2_, respectively, where t_1_ > t_2_. Q_10__O__2_ and Q_10__Lac_ represent the temperature coefficients for changes in QO_2_ and hemolymph lactate, respectively.

### Muscle Lactate Dehydrogenase Kinetics

Each abdominal muscle sample was homogenized (Eurostar Power-B, IKO Labortechnik) on crushed ice at 1,500 rpm for 60 s in 50 mM K_2_HPO_4_ buffer (7:1 v/m, mL g^–1^, unadjusted pH) (modified from Zietara et al., 1996). The homogenate was then centrifuged at 14,000 g for 40 min at 4°C and the supernatant was separated for enzyme kinetic assays. The LDH assays (1 ≤ *N* ≤ 4, pools of samples from 2 to 3 individual crabs) were performed using 100 mL of supernatant diluted from 10 to 2,000 times, depending on condition and species. This assay evaluates the oxidation of NADH to NAD^+^ assuming steady-state conditions such as the linear relationship between NAD^+^ formation and time (Segel, 1976). Controls without added homogenate were conducted to evaluate spontaneous NADH oxidation.

LDH activity was measured continuously, accompanying the oxidation of NADH to NAD^+^ at 340 nm in thermostatted cells (ε_340 nm, pH 6_._9_ = 5,860 mM^–1^cm^–1^; Cary 60 UV-Vis Spectrophotometer, Agilent Technologies) at both 25°C and at the respective FRT, LL_50_, and UL_50_ temperatures of each crab species for up to 5 min. LDH activity was not measured at the LL_50_’s of *Acanthocyclus albatrossis* and *Halicarcinus planatus* owing to the inability of the spectrophotometer to read at these low temperatures (≈-1°C).

For the kinetic assays, up to 14 pyruvate concentrations ranging from 1 μM to 7 mM were used in 80 mM Imidazole-HCl buffer at pH 6.9, containing 150 μM NADH in a final volume of 1 mL (modified from Stillman and Somero, 2001). The pH of the Imidazole buffer was adjusted according to the temperature of each assay. The kinetic parameters, i.e., maximum LDH activity (V_max_, U mg^–1^), the Michaelis-Menten constant for pyruvate (K_m_^Pyr^, mM) and the catalytic coefficient (min^–1^ 10^3^) were calculated using the saturation curve for LDH activity against pyruvate concentration, employing the SigrafW application (Leone et al., 2005). K_m_^Pyr^ describes the enzyme-substrate affinity, representing the pyruvate concentration at which LDH activity is V_max_/2, indicating the ability to form an enzyme-substrate complex. The catalytic coefficient was measured at the FRT and was calculated for 25°C using a Q_10_ of 2, reflecting a measure of the rates of enzyme-substrate formation and dissociation (Segel, 1976; Northrop, 1998) and, thus, the rate constant for transforming pyruvate (substrate) into lactate (product). V_max_ also was calculated for 25°C, using a Q_10_ of 2. One enzyme unit (U) is defined as the amount of LDH that hydrolyzes 1 mmol of NADH per minute. Assays were performed in duplicate aliquots.

Total protein titers in the homogenates were measured following Read and Northcote (1981), using bovine serum albumin as the standard.

### Statistical Analyses

For the intra-specific analyses, the effects of critical thermal limits on the systemic parameters QO_2_, lactate concentration and their respective Q_10_’s, and on the kinetic parameters V_max_ and K_m_^Pyr^, for each species, were evaluated using a one-way Analysis of Variance followed by the Student-Newman-Keuls (SNK) multiple means comparison procedure to detect differences among groups (*P* ≤ 0.05). Data are expressed as the mean ± SEM.

Inter-specific comparisons were conducted based on a molecular phylogenetic analysis of 36 species, performed by a maximum likelihood procedure using the 16S ribosomal gene (Faria et al., 2017), a marker commonly used in phylogenetic studies of decapod Crustacea (e.g., Schubart et al., 2000; Mantelatto et al., 2009a,b; Pileggi and Mantelatto, 2010). Briefly, the phylogenetic analysis was undertaken using the Maximum Likelihood search method (Felsenstein, 1973, 1981) developed in RAxML 7.2.7 (Stamatakis, 2006) and implemented on the CIPRES system, employing the GTR + ∫ + I substitution model (Tavaré, 1986). A rapid bootstrap method (1,000 replicates) (Stamatakis et al., 2008) was used to evaluate topology consistency, only confidence values greater than 50% being considered. The original tree was pruned, maintaining only those species physiologically evaluated here. Branch lengths reflect genetic divergence.

The hypothesis that zoogeographical province may have affected the evolution of the physiological traits at the FRT, LL_50_, or UL_50_ was tested using Phylogenetic Generalized Least Squares (PGLS) models. PGLS assumes that the residual variation among species is correlated owing to their shared ancestry (Grafen, 1989; Garland and Ives, 2000; Lavin et al., 2008), following a hierarchical disposition through time. Since character evolution is mathematically modeled along each branch of the phylogeny, we tested two evolutionary models simultaneously for each PGLS, employing the α parameter. The α-value is a measure of the selection strength that constrains a trait toward an optimum value: unconstrained character evolution (α = 0) is best fitted by the “Brownian motion” evolutionary model, while trait evolution with weakly limited or unlimited minimum and maximum values is best represented by the “Ornstein-Uhlenbeck” (O-U) model of evolution (Revell, 2010). This procedure was performed to ensure the best-adjusted evolutionary correlation for each set of inter-specific data.

Phylogenetic signal, a measure of the tendency of closely related species to resemble each other more than those drawn randomly from a clade, was calculated using Abouheif’s test (Abouheif, 1999; Pavoine et al., 2008). An independent Monte Carlo test was conducted for each trait to provide its Moran’s *I* index and respective P-value. Moran’s *I* varies from −1 to +1, revealing physiological dissimilarities and similarities, respectively, between closely related species (Gittleman and Kot, 1990; Diniz-Filho, 2001).

Overall physiological variability was analyzed using a phylogenetic principal components analysis (pPCA; Jombart et al., 2010). The phylogenetic structure of the multivariate comparative data was obtained by including all measured traits simultaneously (except V_max_ and K_m_^Pyr^ for the sub-Antarctic species where data were unavailable), including microhabitat temperature and both critical thermal limits, for all species. The scores of the two main dimensions (PC_1_ and PC_2_) for each species were then plotted against the phylogeny.

We also tested for the presence of convergent evolutionary events using the SURFACE method (Ingram and Mahler, 2013) to evaluate whether the hypothesis of physiological shifts among the three zoogeographic provinces is more likely than that expected by chance. Comparisons among several simulated O-U models were performed using the stepwise Akaike Information Criterion (AIC) procedure, and all improvements in AIC values were considered.

Comparative analyses were conducted using the R environment (R Development Core Team, 2019), specifically *ape* (Paradis et al., 2004), *nlme* (Pinheiro et al., 2015), *phytools* (Revell, 2012), *adephylo* (Jombart et al., 2010), and SURFACE (Ingram and Mahler, 2013) packages. *P*-values were set at 0.05.

Our findings can be considered free of bias from the species’ vertical positions within their intertidal zones since this parameter had no effect on any physiological trait (pGLS ANOVA, 0.77 ≤ *F* ≤ 3.9, 0.23 ≤ *P* ≤ 0.54). Our characterization of the vertical distributions assumed the five categories distinguished by Stillman and Somero (2000).

## Results

### Thermal Effects on Systemic Metabolism: QO_2_, Hemolymph Lactate and Q_10_

**FIGURE 2:**
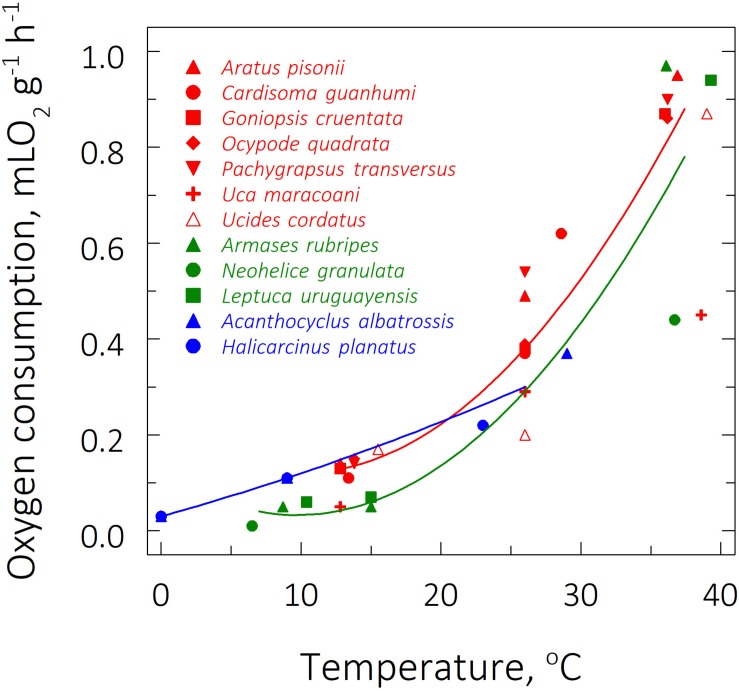
Effect of direct transfer of tropical (red), subtropical (green), and sub-Antarctic (blue) crab species on mass-specific oxygen consumption rate (QO_2_) after 6 h exposure to their respective upper and lower critical thermal limits, from their corresponding field reference temperatures of 26, 15, and 9°C. Data (mL O_2_ g^–1^ h^–1^) are mean values (6 ≤ *N* ≤ 9) and were adjusted to a second degree polynomial curve.

The subtropical species *Neohelice granulata* and *Armases rubripes* exhibited the lowest (0.01 ± 0.00 mLO_2_ g^–1^ h^–1^) and highest QO_2_’s (0.97 ± 0.08 mLO_2_ g^–1^ h^–1^) at their respective LL_50_ (6.5°C) and UL_50_ (36.1°C) (Table 2 and Figure 2). Both these species also showed the most sensitive QO_2_’s (Q_10__O__2_’s of 3.7 and 3.1, respectively). Interestingly, the sub-Antarctic species *Acanthocyclus albatrossis* and *Halicarcinus planatus* at the FRT of 9°C exhibited higher QO_2_’s (≈0.11 mLO_2_ g^–1^ h^–1^) than all the subtropical representatives (*Armases rubripes, N. granulata* and *Leptuca uruguayensis* [≈0.06 mLO_2_ g^–1^ h^–1^]) at the FRT of 15°C (Table 2).

**TABLE 2:** Crab species and their systemic physiological parameters, mass-specific oxygen consumption rate (mL O_2_ g^–1^ h^–1^) and hemolymph lactate concentration (mg L^–1^), measured at their respective field reference temperatures (FRT) and lower (LL_50_) and upper (UL_50_) critical thermal limits.

**Species**	**O_2_ consumption**			**Hemolymph lactate**			**Temperature**
	**(mLO_2_ g^–1^ h^–1^)**			**(mg L^–1^)**			**coefficients**
	**LL_50_**	**FRT**	**UL_50_**	**LL_50_**	**FRT**	**UL_50_**	**Q_10_O_2_/Q_10__Lac_**
**Brazilian province (Tropical)**							
*Aratus pisonii*	0.14 ± 0.03*	0.49 ± 0.06	0.95 ± 0.22*	62 ± 7	61 ± 2	177 ± 35*	2.2/1.6
*Cardisoma guanhumi*	0.11 ± 0.02*	0.37 ± 0.11	0.62 ± 0.08*	41 ± 6*	174 ± 30	167 ± 38	2.1/1.9
*Goniopsis cruentata*	0.13 ± 0.04*	0.38 ± 0.07	0.87 ± 0.14*	62 ± 6*	153 ± 27	231 ± 26*	2.4/1.8
*Ocypode quadrata*	0.15 ± 0.04*	0.39 ± 0.09	0.86 ± 0.10*	25 ± 3	90 ± 33	172 ± 34	2.1/2.2
*Pachygrapsus transversus*	0.14 ± 0.03*	0.54 ± 0.10	0.90 ± 0.09*	136 ± 19	146	144 ± 50	2.3/1.0
*Uca maracoani*	0.05 ± 0.00*	0.29 ± 0.07	0.45 ± 0.10*	37 ± 4	93 ± 25	137 ± 29	2.5/1.7
*Ucides cordatus*	0.17 ± 0.05	0.20 ± 0.07	0.87 ± 0.13*	54 ± 10	76 ± 31	222 ± 63*	2.1/1.9
	0.13 ± 0.02*	**0.38 ± 0.11**	0.78 ± 0.07*	60 ± 14*	**128 ± 25**	179 ± 14*	**2.3/1.7**
**Argentinian province (Subtropical)**							
*Armases rubripes*	0.05 ± 0.01	0.05 ± 0.01	0.97 ± 0.08*	94 ± 19	95 ± 30	138 ± 29	3.1/1.2
*Neohelice granulata*	0.01 ± 0.00*	0.07 ± 0.02	0.44 ± 0.05*	29 ± 6	21 ± 3	73 ± 19*	3.7/1.4
*Leptuca uruguayensis*	0.06 ± 0.02	0.07 ± 0.01	0.94 ± 0.13*	6 ± 2	10 ± 3	11 ± 2	2.3/1.2
	**0.04 ± 0.02**	**0.06 ± 0.01**	0.78 ± 0.30*	**43 ± 26**	**42 ± 27**	74 ± 37*	**3.0/1.3**
**Magellanic province (Sub-Antarctic)**							
*Acanthocyclus albatrossis*	0.03 ± 0.01*	0.11 ± 0.03	0.37 ± 0.03*	23 ± 2	26 ± 5	1,564 ± 255*	2.5/4.6
*Halicarcinus planatus*	0.03 ± 0.00*	0.11 ± 0.01	0.22 ± 0.03*	19 ± 3*	33 ± 5	747 ± 88*	2.5/5.4
	0.03 ± 0.00*	**0.11 ± 0.00**	0.30 ± 0.11*	21 ± 2*	**30 ± 4**	1,155 ± 409*	**2.5/5.0**

Temperature sensitivities (Q_10_O_2_ and Q_10__*Lac*_) for both traits are also provided. Data are the mean ± SEM (4 ≤ N ≤ 10). *Significantly different from the respective FRT value (ANOVA, SNK, P ≤ 0.05). Bold values are the mean ± SEM for all species in each province.

**FIGURE 3:**
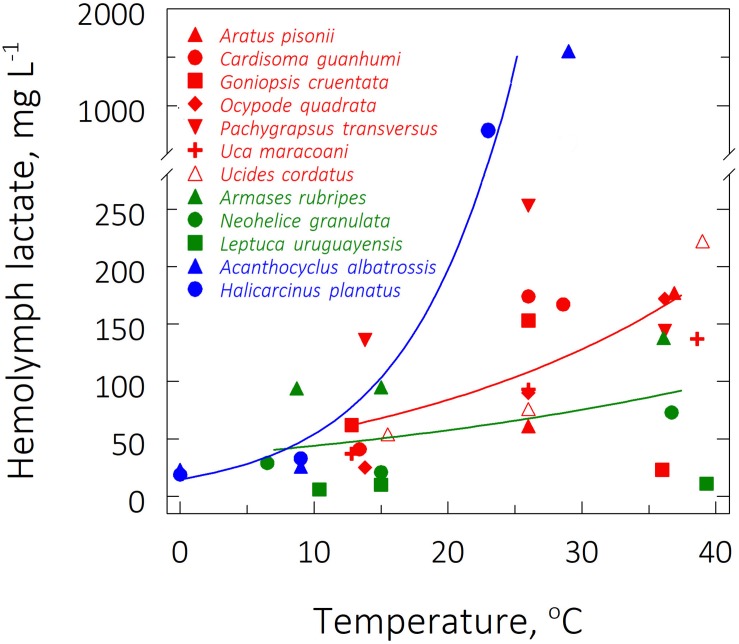
Effect of direct transfer of tropical (red), subtropical (green) and sub-Antarctic (blue) crab species on hemolymph lactate concentration after 6 h exposure to their respective upper and lower critical thermal limits, from their corresponding field reference temperatures of 26, 15, and 9°C. Data (mg L^–1^) are mean values (4 ≤ *N* ≤ 7), and were adjusted to a second degree polynomial curve.

The lowest hemolymph lactate concentration ([Lac]) was found in the subtropical species *Leptuca uruguayensis* (6 ± 2 mg/L) at its LL_50_ of 10.4°C. The sub-Antarctic species *A. albatrossis* showed the highest concentrations (1,564 ± 255 mg/L, respectively) at its UL_50_ of 29°C (Figure 3). Q_10__Lac_ values consistently fell between 1.2 and 1.9, the highest values being found in the sub-Antarctic species, particularly *H. planatus* (5.4) (Table 2).

Critical temperatures affected mean QO_2_ (ANOVA 20 ≤ *F* ≤ 46, *P* ≤ 0.004) and mean hemolymph [Lac] (ANOVA, 16 ≤ *F* ≤ 25, *P* ≤ 0.05) in the tropical and Sub-Antarctic species. These traits increased at the respective mean UL_50_’s of ≈37 and ≈26°C and diminished at the mean LL_50_’s of ≈14°C and ≈0°C, both compared to the FRT’s of 26 and 9°C, respectively (SNK, *P* ≤ 0.05) (Table 2). However, for the subtropical representatives, the mean QO_2_’s and mean hemolymph [Lac] were similar when comparing these rates at the FRT of 15°C and the mean LL_50_ of ≈9°C (SNK, *P* = 0.23), demonstrating that metabolism was unchanged during cooling. Values were higher, however, at the mean UL_50_ of ≈37°C (SNK, *P* ≤ 0.05).

### Evolutionary and Comparative Patterns

**FIGURE 4:**
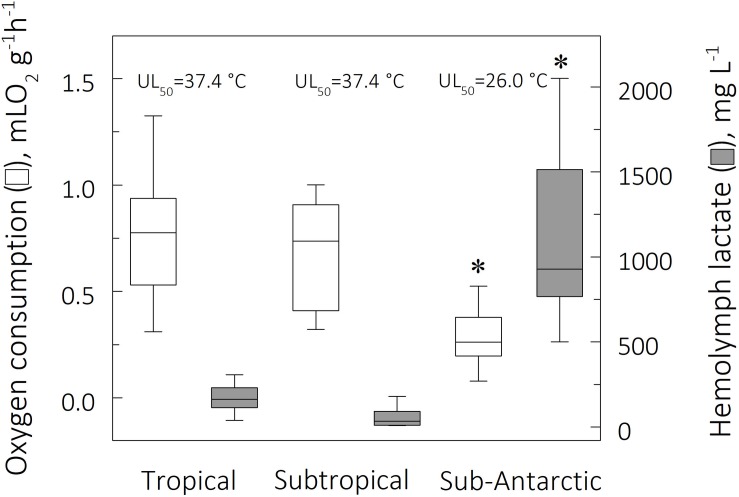
Mean mass-specific oxygen consumption (QO_2_, mL O_2_ g^–1^ h^–1^) and mean hemolymph lactate concentrations (mg L^–1^) for tropical, subtropical and sub-Antarctic crab species at their respective UL_50_, after 6 h direct transfer from their corresponding field reference temperatures of 26, 15, and 9°C. The horizontal line within each box indicates the median QO_2_ while the box boundaries show the interquartile values. Whiskers indicate the lowest and highest QO_2_’s (range). Specimens (12 ≤ *N* ≤ 41 per species) for all species sampled from each zoogeographical province were used to provide the plots. *Significantly different from tropical and subtropical species (phyHolm-Bonferroni, *P* ≤ 0.05).

Zoogeographical province affected the evolution of QO_2_ under all three thermal regimes (pGLS ANOVA, 9 ≤ *F* ≤ 18, *P* ≤ 0.02, 0.14 ≤ α ≤ 21). At the FRT of 26°C, tropical species showed a higher mean QO_2_ (0.38 ± 0.11 mLO_2_ g^–1^ h^–1^) than subtropical (0.06 ± 0.01 mLO_2_ g^–1^ h^–1^1) and sub-Antarctic (0.11 ± 0.00 mLO_2_ g^–1^ h^–1^) species, which also differed between their respective FRT’s of 15 and 9°C, respectively (phyHolm-Bonferroni, *P* = 0.05) (Table 2). Further, mean QO_2_’s at the critical temperature limits showed different evolutionary patterns. Mean QO_2_ at the mean UL_50_ was lower in the sub-Antarctic species (0.30 ± 0.11 mLO_2_ g^–1^ h^–1^) than in the tropical (0.88 ± 0.13 mLO_2_ g^–1^ h^–1^) and subtropical (0.78 ± 0.3 mLO_2_ g^–1^ h^–1^) representatives (phyHolm-Bonferroni, *P* = 0.02) (Figure 4). However mean QO_2_ was similar (≈0.07 mLO_2_ g^–1^ h^–1^) for all provinces at the mean LL_50_ (phyHolm-Bonferroni, *P* ≥ 0.68). The evolution of Q_10_ was not driven by thermal regime (pGLS ANOVA, *F* ≤ 3.3, *P* ≥ 0.1), showing a mean sensitivity of 2.5 (Table 2).

In contrast, zoogeographical province affected the evolution of hemolymph [Lac] only at the mean UL_50_ (pGLS ANOVA, *F* = 7.9, *P* ≤ 0.05, 44 ≤ α ≤ 47). Mean hemolymph [Lac] for the sub-Antarctic species was greater (1,155 ± 409 mg L^–1^) than those of the tropical (179 ± 14 mg L^–1^) and subtropical (74 ± 37 mg/L) representatives (phyHolm-Bonferroni, *P* ≤ 0.05), the values for the latter groups being similar (phyHolm-Bonferroni, *P* ≥ 0.05) (Table 2 and Figure 4). Mean hemolymph [Lac] at the mean LL_50_ (49 ± 11 mg L^–1^, all species) and at the FRT (90 ± 21 mg L^–1^, all species) were similar among provinces (phyHolm-Bonferroni, *P* ≥ 0.05). Mean Q_10_’s for hemolymph lactate mirrored this similarity (pGLS ANOVA, 7.5 ≤ *F* ≤ 7.8, *P* ≤ 0.05), being greater in the sub-Antarctic species (3.9 ± 1.5) than in the tropical (1.7 ± 0.1) and subtropical species (1.3 ± 0.1).

### Thermal Effects on Muscle LDH Kinetics: V_max_, K_m_^Pyr^ and Catalytic Coefficient

**FIGURE 5:**
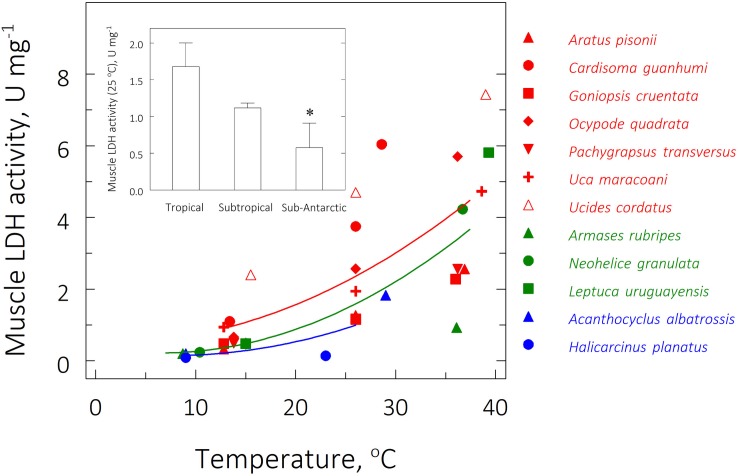
Effect of direct transfer of tropical (red), subtropical (green), and sub-Antarctic (blue) crab species on muscle lactate dehydrogenase (LDH) activity (V_max_, U mg^–1^) after 6 h exposure to their respective upper and lower critical thermal limits from their corresponding field reference temperatures (FRT) of 26, 15, and 9°C. Data are mean values (2 ≤ *N* ≤ 3, pools of 3 individuals each) and were adjusted to a second degree polynomial curve. V_max_ was measured continuously in duplicate aliquots at the same experimental temperatures. Activities could not be measured for the sub-Antarctic species at their lower critical thermal limits. *Insert*: Mean (± SEM) muscle LDH activity at 25°C for all species from each province (2 ≤ *N* ≤ 3, pools of 3 individuals each); muscle samples taken at the FRT of each species. *Significantly different from tropical crab species (phyHolm-Bonferroni, *P* ≤ 0.05).

The highest V_max_ was 7.43 ± 0.39 U mg^–1^ for the tropical species *Ucides cordatus* at its UL_50_ of 39.0°C while the lowest activity was 0.09 ± 0.00 U mg^–1^ in the sub-Antarctic species *Halicarcinus planatus* at the FRT of 9.0°C (Table 3 and Figure 5). At the zoogeographical province level, the mean V_max_’s for the tropical and subtropical species were strongly sensitive to critical temperature exposure: V_max_’s were higher at the mean UL_50_ and lower at the mean LL_50_ (ANOVA; 11.5 ≤ *F* ≤ 15.2; *P* ≤ 0.01). The mean V_max_ for the sub-Antarctic species was thermally insensitive (ANOVA; *F* = 1.0; *P* = 0.4). At an extrapolated temperature of 25°C, mean V_max_ values were affected by zoogeographical province (pGLS ANOVA, *F* = 6.0, *P* = 0.05, α = 16.9) with calculated values of 1.7 ± 0.3, 1.1 ± 0.1 and 0.6 ± 0.3 U mg^–1^ for the tropical, subtropical and sub-Antarctic species, respectively (Table 3 and Figure 5, *insert*).

**TABLE 3:** Crab species and their kinetic parameters, LDH activity (V_max_, U mg*^–^*^1^) and K_m_^Pyr^ (mM), measured at their respective field reference temperatures (FRT) and lower (LL_50_) and upper (UL_50_) critical thermal limits.

**Species**	**LDH activity**			**K_m_^Pyr^**			**Catalytic coefficient**
	**(V_max_, U mg^–1^)**			**(mM)**			**(min^–1^ 10^3^)**
	**LL_50_**	**FRT**	**UL_50_**	**LL_50_**	**FRT**	**UL_50_**	**25°C**
**Brazilian province (Tropical)**							
*Aratus pisonii*	0.33 ± 0.02*	1.27 ± 0.10	2.56 ± 0.04*	0.06 ± 0.00*	0.11 ± 0.00	0.20 ± 0.00*	10.6
*Cardisoma guanhumi*	1.11 ± 0.17*	3.75 ± 0.16	6.04 ± 0.03*	0.22 ± 0.03*	0.17 ± 0.01	0.61 ± 0.01*	20.2
*Goniopsis cruentata*	0.48 ± 0.13	1.15	2.28	0.06 ± 0.01	0.10	0.2	10.6
*Ocypode quadrata*	0.66 ± 0.06*	2.57 ± 0.11	5.70 ± 1.81	0.08 ± 0.00*	0.15 ± 0.02	0.36 ± 0.04	16.7
*Pachygrapsus transversus*	0.49 ± 0.12*	1.17 ± 0.09	2.56 ± 0.04*	0.22 ± 0.00	0.20 ± 0.01	0.29 ± 0.00*	5.4
*Uca maracoani*	0.94 ± 0.04*	1.94 ± 0.09	4.73 ± 0.31*	0.37 ± 0.01	0.49 ± 0.05	0.57 ± 0.09	3.5
*Ucides cordatus*	2.4 ± 0.4*	4.70 ± 0.10	7.43 ± 0.39*	0.17 ± 0.03	0.17 ± 0.01	0.34 ± 0.01*	26.5
	0.92 ± 0.27*	**2.36 ± 0.53**	4.47 ± 0.77*	**0.17 ± 0.0**	**0.20 ± 0.05**	0.37 ± 0.06*	**13.4 ± 3.1**
**Argentinian province (Subtropical)**							
*Armases rubripes*	0.20 ± 0.05	0.50 ± 0.08	0.93 ± 0.12*	0.14 ± 0.02	0.15 ± 0.01	0.16 ± 0.02	7.0
*Neohelice granulata*	0.24 ± 0.05	0.47 ± 0.09	4.23 ± 0.30*	0.37 ± 0.02	0.32 ± 0.03	0.37 ± 0.01	3.0
*Leptuca uruguayensis*	n.e.	0.48 ± 0.10	5.81 ± 0.11*	0.15 ± 0.02	0.16 ± 0.00	0.66 ± 0.01*	5.8
	0.22 ± 0.02*	**0.48 ± 0.02**	3.66 ± 1.44*	**0.22 ± 0.06**	**0.21 ± 0.1**	**0.40 ± 0.15**	**5.3 ± 1.2**
**Magellanic province (Sub-Antarctic)**							
*Acanthocyclus albatrossis*	n.e.	0.20 ± 0.03	1.83 ± 0.35*	n.e.	0.30 ± 0.00	0.32 ± 0.00	2.1
*Halicarcinus planatus*	n.e.	0.09 ± 0.00	0.14 ± 0.01*	n.e.	0.50 ± 0.00	0.37 ± 0.03*	0.61
		**0.15 ± 0.05**	**0.99 ± 0.85**		**0.40 ± 0.10**	**0.35 ± 0.03**	**1.4 ± 0.7**

Catalytic coefficient (min^–1^ 10^3^) is calculated for 25°C, using a Q_10_ of 2 estimated from muscle samples at the FRT of each species. Data are the mean ± SEM (1 ≤ N ≤ 3, pools of 2–3 individuals per sample). *Significantly different from the respective FRT value (ANOVA, SNK, P ≤ 0.05). n.e., not estimated. Bold values are the mean ± SEM for all species in each province.

**FIGURE 6:**
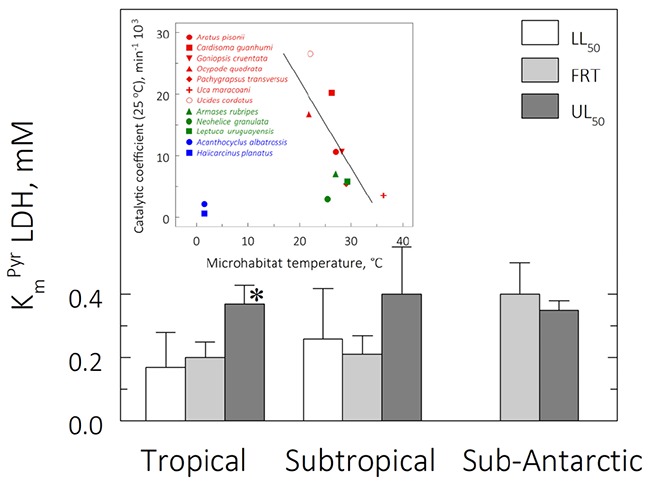
Michaelis-Menten constants (K_m_^Pyr^, mM) for muscle lactate dehydrogenase (LDH) in tropical, subtropical and sub-Antarctic crab species at their respective lower (LL_50_) and upper (UL_50_) critical thermal limits after 6 h direct transfer from the corresponding field reference temperatures (FRT) of 26, 15, and 9°C. Data are the mean ± SEM (2 ≤ *N* ≤ 3, pools of 3 individuals each). LDH activity was measured continuously in duplicate aliquots at the same experimental temperatures. Measurements could not be made for the sub-Antarctic species at their lower critical thermal limits. *Significantly different from tropical and subtropical crabs (phyHolm-Bonferroni, *p* ≤ 0.05). *Insert*: Catalytic coefficient (min^–1^ 10^3^) calculated for 25°C, assuming a Q_10_ of 2, plotted against the microhabitat temperature.

Mean K_m_^Pyr^ for the subtropical and sub-Antarctic crab species were not affected by exposure at their critical thermal limits (ANOVA, 0.62 ≤ *F* ≤ 0.95; *P* ≥ 0.43) (Table 3). However, mean K_m_^Pyr^ for the tropical species was affected by exposure at the mean UL_50_ (ANOVA, *F* = 4.6, *P* ≤ 0.05; SNK, *P* ≤ 0.05). The mean K_m_^Pyr^ for the three provinces under all thermal regimes was 0.3 mM (Figure 6). In contrast, the catalytic coefficients calculated for 25°C (3.0–26.5 min^–1^ 10^3^; Figure 5, insert) did regress on microhabitat temperatures (pGLS, −0.62 ≤ slope ≤ −0.64, 5.4 ≤ *F* ≤ 8.8, *P* ≤ 0.05, α = 22.9), when the sub-Antarctic species *A. albatrossis* and *H. planatus* are excluded from the analysis (see also Table 3).

### Evolutionary and Comparative Patterns

Zoogeographic province affected the evolution of muscle LDH activity at the FRT (pGLS ANOVA, 6.1 ≤ *F* ≤ 7.2, *P* ≤ 0.03, α = 11.5). Tropical species showed a mean V_max_ (2.4 ± 0.5 U mg^–1^) greater than that of the subtropical (0.5 ± 0.0 U mg^–1^) and sub-Antarctic (0.2 ± 0.1 U mg^–1^) species (phyHolm-Bonferroni, *P* ≤ 0.05) (Table 3). However, there was no effect of province on V_max_ at either mean critical limit (pGLS ANOVA, 1.1 ≤ *F* ≤ 2.6, *P* ≥ 0.06). Tropical (0.9 ± 0.3 U mg^–1^) and subtropical representatives (0.2 ± 0.0 U mg^–1^) showed similar mean V_max_’s at their mean LL_50_’s (phyHolm-Bonferroni, *P* ≥ 0.14) and at their mean UL_50_’s (4.5 ± 0.8 U mg^–1^ and 3.7 ± 1.4 U mg^–1^, respectively) (phyHolm-Bonferroni, *P* ≥ 0.14).

The evolution of K_m_^Pyr^ was not affected by province under any thermal regime (pGLS ANOVA, 0.009 ≤ *F* ≤ 2.5, *P* ≥ 0.14) (Figure 6). Mean K_m_^Pyr^ was 0.2 mM at the mean FRT, 0.21 mM at the mean LL_50_ and 0.37 mM at the mean UL_50_. However, catalytic coefficient was associated with microhabitat temperature (pGLS ANOVA; −0.62 ≤ slope ≤ −0.64, 5.4 ≤ *F* ≤ 8, *P* ≤ 0.05, ΔAIC ≤ 2, α = 22.9), excluding the two outliers from the Magellanic province (Figure 6, insert).

### Phylogenetic Signal and Multi-Variate/Dimensional Patterns

With regard to the upper critical limit (UL_50_), all traits, i.e., Q_O__2_, [Lac], V_max_ and K_m_^Pyr^, as well as UL_50_ itself, showed significant phylogenetic signal (*I* > 0.28, P < 0.05), thus their inter-specific variability exhibits a phylogenetic pattern. However, all other traits regarding exposure to FRT and LL_50_ were unrelated to phylogeny (−0.22 ≤ *I* ≤ 0.19, *P* ≥ 0.05).

**FIGURE 7:**
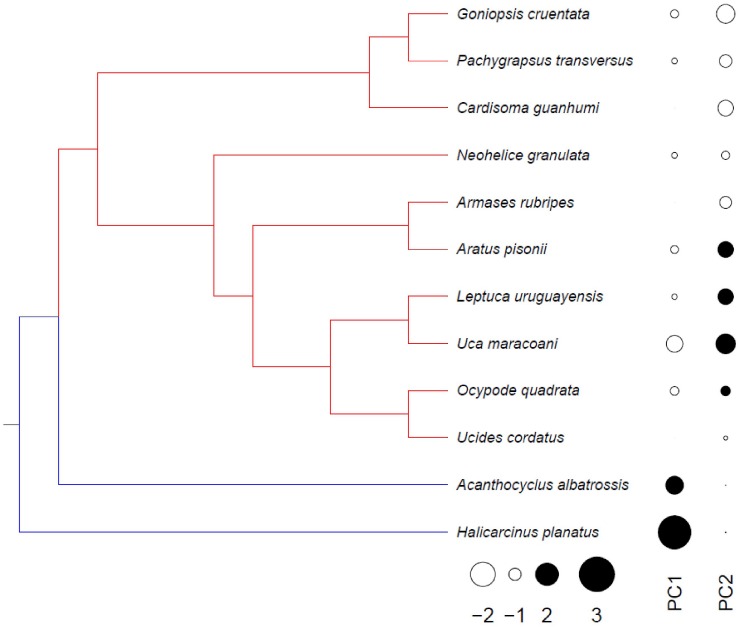
Overall physiological evolution of thermal tolerance, grouping systemic and kinetic traits in intertidal crabs distributed from northern Brazil to southern Patagonia, mapped onto a phylogeny modified from Faria et al. (2017). Different colors represent adaptive peaks identified using the SURFACE method: the first optimum (blue) was detected for the sub-Antarctic species while the second (red) was found in the tropical and subtropical representatives. Using a phylogenetic PCA, the first two dimensions accounted for 54.3% of total physiological variance, specifically 38.1% for PC_1_ and 16.2% for PC_2_. The scores of both dimensions follow the same phylogenetic pattern as the adaptive peaks. Microhabitat temperature, LL_50_ and UL_50_ were the most important parameters in both analyses (*R* ≥ 0.30 for pPCA, −14 < AIC < −8 for SURFACE).

Phylogenetic principal components analysis, grouping all traits, revealed that the first two dimensions accounted for 54.3% of the total physiological variance, specifically 38.1% for PC_1_ and 16.2% for PC_2_ (pPCA, Figure 7): PC_1_ is more related to the sub-Antarctic species while PC_2_ to the tropical/subtropical clade. This scenario is in agreement with the two adaptive peaks detected in the phylogeny: (i) at the root, and maintained in *A. albatrossis* and *H. planatus*; and (ii) a shift at the outset of the tropical and subtropical species (SURFACE analysis, Figure 7). Microhabitat temperature, LL_50_ and UL_50_ were the traits that most strongly correlated with the more relevant pPCA dimension (*R* ≥ 0.30), and also contributed most to the adaptive peaks (−14 < AICc < −8) as evaluated using SURFACE.

## Discussion

We found that tropical and subtropical intertidal crab species showed similar subcellular and organismic responses to acute thermal stress, differing from their sub-Antarctic relatives. Oxygen consumption (QO_2_) and hemolymph lactate ([Lac]) increased with increasing temperature in all species from the three zoogeographical provinces, revealing an apparent trade-off with the evolution of upper critical thermal limit (UL_50_). While maximum LDH activity (V_max_) also increased with temperature, K_m_^Pyr^ tended to remain constant, whereas the catalytic coefficient is driven by micro-habitat temperature in most cases. Interestingly, all physiological traits at the UL_50_ have evolved in a phylogenetic fashion, while the others linked to LL_50_ and FRT were more plastic. Overall metabolic evolution at both critical limits has taken place by two independent adaptive pathways in the sub-Antarctic lineages, however one adaptive peak has been inherited by the northernmost species. These physiological patterns and evolutionary history appear to reflect the species’ ability to function at higher temperatures, together with a lesser capacity to confront lower temperatures, since the crabs were likely acclimatized to the southern summer. The discussion that follows should be considered in this light.

### Systemic Metabolism

The systemic metabolism complied with the paradigm that higher temperatures lead to greater energy demands through increased QO_2_ and [Lac]. The highest values for these parameters were seen at the UL_50_ while the lowest values occurred at the LL_50_, both compared to the FRT. At the FRT, the tropical crab species exhibited a mean QO_2_ 6-fold greater than the mean value for the subtropical crabs, and 3-fold greater than the sub-Antarctic representatives. The same phenomenon has occurred in the Echinodermata, Annelida, other Crustacea, and Mollusca: warmer water species show higher rates of oxygen consumption than do colder water species when measured at their respective usual environmental temperatures (Fox, 1936), reaching up to 10-fold greater in crustaceans from tropical waters than in polar species (Scholander et al., 1953). Further, the sub-Antarctic crab species showed a 2-fold higher QO_2_ at the FRT of 9°C than did the subtropical crabs at the FRT of 15°C, which reveals temperature compensation of aerobic metabolism in cold regions, an adaptive peak probably maintained by strong stabilizing selection as revealed by the higher α-value.

The evolution of temperature compensation of aerobic capacity as observed here may result from the differential regulation of hemolymph Mg^2+^ as seen in some decapod crustaceans (Frederich et al., 2000; Wittmann et al., 2010, 2012) or may be due to selective pressure on cellular energy demand, as demonstrated in fish (Peck et al., 2002; Pörtner, 2002; Morley et al., 2009). Hemolymph Mg^2+^ hypo-regulation is associated with higher activity levels in decapods from low temperatures since higher Mg^2+^ concentrations diminish activity by inhibiting Ca^2+^ influx across neuronal membranes, slowing down neuromuscular transmission and decreasing muscle contraction (Parnas et al., 1994; Dunn and Mercier, 2003). Elevated activity levels in decapods from naturally or experimentally cold temperatures are due to fairly high heart rates, ventilation frequencies and oxygen consumption, as a consequence of lower Mg^2+^ levels (Frederich and Pörtner, 2000). Increased mitochondrial densities also contribute to elevated aerobic metabolism in fish acclimated to cold temperatures revealing ultrastructural adjustments (Johnston and Maitland, 1980; Tyler and Sidell, 1984). Both hypotheses, in combination or independently, may explain the higher QO_2_ values seen in the sub-Antarctic species.

With regard to phylogenetic signal for oxygen consumption, closely related species do not show similar QO_2_’s at the FRT and LL_50_, which is also supported by the effect of zoogeographical province on QO_2_ under both thermal regimes together with a significant effect of selection strength (17 ≤ α ≤ 20). Given the physical dependence of QO_2_ on the kinetic energy of the molecules, this scenario suggests adaptive evolution strongly driven by thermal regime, regardless of the phylogenetic position of each species. To illustrate, the fiddler crabs sampled, i.e., the tropical species *U. maracoani* and the subtropical species *L. uruguayensis*, although closely related, exhibit distinct QO_2_’s under their natural temperature conditions and on cold challenge. Conversely, aerobic metabolism at the UL_50_ is similar among closely related species, also reinforced by the absence of a province effect, signifying that inter-specific variability in QO_2_ at higher temperatures is constrained by shared inheritance, regardless of a significant effect of thermal regime. The maximum aerobic capacities at higher temperature challenge vary among species in a phylogenetic fashion.

With respect to anaerobic metabolism, mean [Lac] in the subtropical crab species did not vary as a function of their respective FRT and LL_50_, while the tropical and sub-Antarctic crabs showed a reduction of ≈50 and 70%, respectively, in mean [Lac] at the mean LL_50_ compared to FRT. Since the subtropical species encounter a eurythermic environment owing to the mixing of warm water from the Brazilian Current with colder water from the Malvinas Current (Boschi, 2000a), their stable anaerobic metabolism suggests the capability for cold acclimatization and energy production even during rapid temperature changes such as might occur during exposure at low tide. In contrast, the tropical and sub-Antarctic crabs hail from more stenothermic provinces, dominated respectively by the South Equatorial Current and by sub-Antarctic waters (Boschi, 2000a; Thurman et al., 2013), suggesting that rapid cooling effects are more pervasive and more able to reduce anaerobic energy production. However, species from all three zoogeographical provinces showed an increase in mean [Lac] during exposure at their UL_50_ compared to the FRT. Mean [Lac] was 1.4- and 1.8-fold higher at the mean UL_50_ of ≈39°C for the tropical and subtropical species, respectively, but 17-fold higher at the mean UL_50_ of 26°C for the sub-Antarctic species, revealing a stronger effect of higher temperatures in species acclimatized to a colder province. The lack of mobilization of lactate at the lower critical limits is possibly due to a lower cellular energy demand, which is compatible with the lower kinetic energy of the molecules.

The high [Lac] at the upper critical temperatures indicates recruitment of anaerobiosis owing to high tissue energy demand, while the high QO_2_’s suggest some cardiorespiratory capability in supplying tissues with oxygen. For example, the spider crab *Maja squinado* shows changes in [Lac] in some tissues in tandem with a reduction in aerobic capacity. High (33°C) and low (0°C) temperatures provoke decoupling between O_2_ supply and demand, since mitochondrial O_2_ consumption is consistent with oxygen delivery by the cardiorespiratory system (Frederich and Pörtner, 2000). Further, in decapod crustaceans subjected to hypoxia or exercise, lactate affects hemocyanin affinity for oxygen, increasing oxygen extraction by the gills and transport by the hemolymph to the tissues as seen in the brachyuran crabs *Cancer*, *Carcinus*, and *Hyas* (Truchot, 1980; Morris and Bridges, 1989), *Homarus* lobsters (Nies et al., 1992) and *Macrobrachium* shrimps (Ern et al., 2013).

Since aerobic and anaerobic metabolism are both linked to the energy supply of a biological system (see Pörtner et al., 2017), the lower mean UL_50_ for the sub-Antarctic crabs (≈26°C) compared to the mean UL_50_ for the tropical and subtropical species (≈37°C) may reflect a mismatch between aerobic and anaerobic energy production at the upper critical limits. In fact, under high thermal challenge, the sub-Antarctic species have evolved a lower aerobic metabolism (0.4-fold), albeit circumvented by an augmented anaerobic capacity (9.1-fold) when compared to the mean values for the tropical and subtropical species (Figure 4). Since aerobic capacity ensures higher ATP production (potentially up to 38 ATP molecules per glucose molecule) than does anaerobic metabolism (only 2 ATP molecules), the lower energy budget available to the sub-Antarctic species could impair overall cellular activities such as anti-oxidant defenses and heat-shock responses, reducing thermal tolerance. The acute stress encountered at the upper critical temperatures constrains aerobic capacity and shifts the energy generating system to a predominantly anaerobic metabolism in crabs from colder climates, suggesting an evolutionary metabolic trade-off during thermal niche diversification.

### LDH Kinetics

For each zoogeographical province, mean V_max_ tends to become reduced at the LL_50_ and to increase at the UL_50_, both compared to the respective FRT. Interestingly, at the fixed temperature of 25°C, there was an effect of zoogeographical province: mean V_max_ for the sub-Antarctic species was 3-fold lower than that for tropical species. This tendency for lower activities at the same temperature for species from colder regions contrasts with previous data for tropical/subtropical and Antarctic fish (Kawall et al., 2002) that show thermal compensation of LDH specific activity. In this case, brain LDH activity was up to 2.5-fold higher in the Antarctic species, while muscle LDH activities were similar between the two thermal groups, both measured at the same temperature of 10°C (Kawall et al., 2002).

The absence of thermal compensation in LDH activity in the intertidal, neotropical crabs evaluated here corroborates findings for another intertidal decapod clade distributed from Northern to Central America, anomuran crabs *Petrolisthes*. No effect of maximum habitat temperature was seen on the evolution of LDH thermal stability in 22 congeneric species sampled over a ≈35° range of latitude, suggesting that the diversity of LDH stabilities also is not adaptive (Stillman and Somero, 2001). Further, similar stabilities at maximum temperatures for some sibling *Petrolisthes* groups were suggested, which is reinforced here for neotropical crabs, owing to the phylogenetic signal seen for V_max_ at UL_50_. Although maximum and critical temperatures, respectively, do not constitute a selective pressure on the evolution of LDH stability (anomurans) and activity (brachyurans), the inter-specific variability in these mechanistic bases demonstrates the importance of ancestral inertia in the evolutionary history of different intertidal decapod lineages.

While V_max_ does not suggest LDH thermal compensation when comparing activities at the same temperature, the catalytic coefficient (also at 25°C) correlated negatively with micro-habitat temperatures (MHT). Species sampled from higher MHT tend to have lower catalytic coefficients and vice-versa (except for the sub-Antarctic species *A. albatrossis* and *H. planatus*). In fact, the lower MHT values (21–26°C) show a mean catalytic coefficient 2.5-fold higher than the mean value for the species from warmer habitats (28–36°C), which means that species from colder MHT’s show more flexible LDH molecules, while conversely, those from warmer habitats show more rigid enzymes. In contrast, mean K_m_^Pyr^ tends to be constant (≈0.27 mM), reflecting retention of LDH affinity for pyruvate and, consequently, its binding efficiency.

The temperature compensation of LDH activity has evolved through the transformation of catalytic efficiency, which is in agreement with maintenance of binding capacity for pyruvate (see Hochachka and Somero, 2002). Such a biochemical trade-off has been documented for several fish species from various thermal niches, such as *Sphyraena* barracudas (Holland et al., 1997), *Chromis* Pacific fish (Johns and Somero, 2004) and four families of Antarctic notothenioids (Fields and Somero, 1998). The maintenance of the LDH binding property demonstrates conservation of the catalytic site. Variability in enzyme efficiency results from alterations to the amino acid sequence in regions that undergo conformational changes during formation of the enzyme-substrate complex (see Hochachka and Somero, 2002). The reasons why LDH in tropical species has lost its affinity (higher K_m_^Pyr^) during exposure at UL_50_, and why sub-Antarctic crabs have not retained a higher catalytic efficiency at their natural MHT, remain conjectural and require investigation at the molecular level.

Overall physiological evolution, when considered simultaneously for all systemic and kinetic traits, including MHT and both critical limits, consists of two steps. The first took place at the outset of the present crab phylogeny, and the second at the outset of the tropical + subtropical lineage, owing to the shift between the two main dimensions, PC_1_ for the sub-Antarctic species and PC_2_ for the tropical and subtropical crabs (Figure 7). Interestingly, these findings are in agreement with the two adaptive peaks detected in this crab phylogeny: one at the root, and maintained in *A. albatrossis* and *H. planatus*, and another at the outset of the tropical and subtropical species. However, since the sub-Antarctic *A. albatrossis* and *H. planatus* are a paraphyletic grouping, also belonging to different eubrachyuran families (Bellidae and Hymenosomatidae, respectively, see Faria et al., 2017), the adaptive peak revealed here should be interpreted as the result of adaptive convergence. The adaptive peaks detected using SURFACE are also in concert with most evolutionary associations detected using PGLS since they were better adjusted by the O-U model, with higher selection strengths that limit minimum and maximum physiological capabilities. The reason why the most important traits that contribute to this macroevolutionary landscape are the thermal niche, and the upper and lower critical thermal limits, may depend on their underlying biological organization, since such traits integrate lower hierarchical levels, including lesser physiological differences, following the synergistic principle of the “part-whole” relationship.

In summary, the metabolic physiology of crabs living at the edge of their thermal windows accompanies the asymmetry of their critical limits. The extension of life to higher temperatures depends on a putative trade-off between the evolution of aerobic and anaerobic metabolism with respect to energy supply, while temperature compensation of kinetic performance is driven by thermal habitat, as revealed by the LDH affinity/efficiency equilibrium. In fact, shared inheritance and the thermal environment both affect the overall physiological history of these intertidal crabs, revealing that certain evolutionary transformations have arisen both in warmer and in colder climes, particularly at higher levels of biological organization and phylogenetic diversity.

## Data Availability Statement

The datasets generated for this study are available on request to the corresponding author.

## Ethics Statement

Crab sampling in Brazil was authorized by the Ministry for the Environment (MMA/ICMBio permit #29594-8 to JM), and in Argentina by the Ministry for the Environment, Sustainable Development and Climate Change of Tierra del Fuego (permit #0116/2014 to MR).

## Author Contributions

SF and JM contributed to the conceptualization, the data curation, the project administration, the funding acquisition, and the visualization. SF, ML, AZ, FT, MR, AB, and JM contributed to the methodology, writing, review, and the editing. SF contributed to the software, writing, and the initial draft. SF, ML, and JM contributed to the formal analysis. SF, ML, FT, MT, and JM contributed to the investigation. SF, AB, FT, MR, and JM contributed to the resources. JM contributed to the supervision.

## Conflict of Interest

The authors declare that the research was conducted in the absence of any commercial or financial relationships that could be construed as a potential conflict of interest.
